# Clinical association and potential molecular mechanisms of neonatal sepsis and necrotizing enterocolitis

**DOI:** 10.3389/fmolb.2025.1662343

**Published:** 2025-10-02

**Authors:** Xue Liu, Wenqiang Sun, Jingtao Bian, Yihui Li, Xinyun Jin, Xueping Zhu

**Affiliations:** ^1^ Department of Neonatology, Children’s Hospital of Soochow University, Suzhou, China; ^2^ Suzhou Medical College, Soochow University, Suzhou, China

**Keywords:** neonatal sepsis, necrotizing enterocolitis, neutrophil chemotaxis, diagnostic biomarkers, transcriptional regulatory network

## Abstract

**Background:**

Necrotizing enterocolitis (NEC) is a severe intestinal disease affecting premature infants, with mortality rates of 20%–30%. Clinical studies have shown that neonatal sepsis (NS) is an independent risk factor for NEC; however, the shared molecular mechanisms and diagnostic biomarkers between these two conditions remain poorly understood. This study aims to explore the shared molecular mechanisms underlying the association between NS and NEC and to identify potential diagnostic biomarkers.

**Methods:**

This study combines clinical cohort analysis with transcriptomic analysis. First, we enrolled 74 NEC infants and 74 gestational age/birth weight-matched controls from Children’s Hospital of Soochow University and quantified the association between NS and NEC using logistic regression analysis. Second, we jointly analyzed transcriptome data from NS (GSE25504) and NEC (GSE46619) datasets to screen for overlapping differentially expressed genes (DEGs) and constructed a protein-protein interaction (PPI) network to identify hub genes. Subsequently, the diagnostic efficacy of core genes was evaluated using independent validation cohorts (GSE297483 and GSE69686). Finally, a transcription factor-mRNA regulatory network was constructed using the TRRUST database to explore the underlying regulatory mechanisms.

**Results:**

Clinical association analysis showed a significantly increased risk of NEC in NS infants (*OR* = 3.02, *P* = 0.002). Infants in the NEC group had significantly higher systemic inflammatory markers and a higher incidence of sepsis (60.81% vs. 33.78%) compared to the control group. Mechanistic studies identified 70 co-directional overlapping DEGs, with 69 upregulated and 1 downregulated. These genes were significantly enriched in neutrophil chemotaxis and *IL-17* signaling pathways (*P* < 0.05). Further investigation identified *FPR1*, *S100A12*, and *CSF3R* as potential biomarkers involved in immune response and inflammatory processes. External validation showed moderate diagnostic performance, with areas under the curve (AUCs) ranging from 0.723 to 0.813. Transcriptional regulation analysis revealed that transcription factors including *SPI1, NFKB1,* and *JUN* were identified as potential regulators of inflammatory genes.

**Conclusion:**

This study suggests that neonatal sepsis may serve as a risk factor for NEC development through shared inflammatory pathways involving *FPR1*, *S100A12*, and *CSF3R*. These genes demonstrated diagnostic potential across both conditions and appear to mediate inflammatory processes involving immune cell recruitment. While these findings suggest new directions for early identification in high-risk infants, further clinical validation is necessary to confirm therapeutic implications.

## 1 Introduction

Necrotizing enterocolitis (NEC) is a severe intestinal disease threatening premature infants, with pathogenesis linked to intestinal barrier damage, dysbiosis, and abnormal immune activation (Hackam and Sodhi, 2022; Kamble et al., 2024). Clinical manifestations range from mild symptoms to severe complications including sepsis and death (Barge et al., 2025). Despite medical advances, NEC incidence and mortality remain high, particularly among extremely low birth weight infants. Clinical observations indicate that neonatal sepsis (NS) frequently accompanies NEC development, with retrospective studies suggesting NS may serve as a risk factor (Modrzejewska and Bosy-Gąsior, 2023). This suggests a potential relationship between NS and NEC. However, the molecular mechanisms underlying their association remain unclear (Wang et al., 2023). Current NEC diagnosis relies on nonspecific inflammatory markers and imaging examinations. These markers lack specificity and often elevate only in late disease stages, potentially causing delayed diagnosis (Agakidou et al., 2020; Howarth et al., 2022). Therefore, there is need for biomarkers with both mechanistic specificity and diagnostic sensitivity.

Existing research indicates NS and NEC may share immune dysregulation mechanisms, particularly through Toll-like receptor 4 (TLR4) pathway activation (Gomart et al., 2021; Bethell and Hall, 2023). However, most studies focus on individual diseases or specific pathways, limiting knowledge of cross-disease driving genes and regulatory networks. This may impede understanding of NS-complicated NEC mechanisms and restrict therapeutic strategy development. To address these gaps, our study employs an integrated approach combining clinical analysis with bioinformatics methods. We analyzed datasets for NEC (GSE46619) and NS (GSE25504) to identify overlapping differentially expressed genes (DEGs), constructed protein-protein interaction (PPI) networks, and validated diagnostic potential using independent datasets. We also explored transcription factor-mRNA (TF-mRNA) regulatory networks.

This study aims to investigate NS as a potential NEC risk factor and explore underlying pathogenesis. We seek to identify core pathways and hub genes common to both diseases and evaluate their diagnostic utility (Alba et al., 2017). Our analysis identified *FPR1*, *S100A12*, and *CSF3R* as potential biomarkers showing diagnostic utility in both conditions. The findings may contribute to NEC pathogenesis understanding and suggest targets for future investigation in high-risk infant identification.

## 2 Methods

### 2.1 Clinical data collection of NEC

Clinical data from 74 cases diagnosed with NEC and discharged from the Children’s Hospital of Soochow University between 1 June 2017, and 1 June 2022, were collected. These cases were matched 1:1 with non-NEC cases from the same period based on gestational age (±3 days) and weight (±100 g), resulting in the formation of NEC and non-NEC groups. The retrospective collection of case data included laboratory indicators, maternal pregnancy indicators, basic information of the infants upon admission, comorbidities, and treatment status. Exclusion criteria included: (1) clear presence of genetic metabolic diseases and chromosomal abnormalities; (2) severe congenital structural malformations; (3) refusal by the infant’s guardian to participate in this study. Diagnostic criteria and definitions: NEC diagnosis and staging were based on the modified Bell’s staging criteria (Patel et al., 2020). Diagnoses of SGA, RDS, PDA, and sepsis were referenced from Avery’s Diseases of the Newborn (Mu and Wang, 2022). Early-onset sepsis (EOS) was defined as sepsis occurring within 72 h of birth, while late-onset sepsis (LOS) was defined as sepsis occurring after 72 h of birth (Glaser et al., 2024). To establish the temporal sequence and minimize reverse causality, we restricted the analysis to sepsis episodes that occurred before the onset of NEC; episodes occurring after NEC onset were excluded. Maternal underlying diseases and comorbidities were all clearly diagnosed in the hospital. This study was approved by the Ethics Committee of the Children’s Hospital of Soochow University (Ethics No. 2023CS130) and conforms to the ethical standards of the 1964 Declaration of Helsinki and its later amendments or comparable ethical standards. Informed consent was obtained from the guardians of the infants, who agreed to the use and disclosure of their clinical data.

### 2.2 Bioinformatics data processing

For NS, we selected the GSE25504 dataset, which contains 44 NS samples and 44 healthy control samples derived from infant blood. For NEC, we selected the GSE46619 dataset, including 5 NEC and 5 healthy control samples from intestinal tissues. Both datasets were obtained from the Gene Expression Omnibus (GEO) database (https://www.ncbi.nlm.nih.gov/geo/) of the National Center for Biotechnology Information (NCBI).

The selection of public datasets was guided by the following criteria: (1) human neonatal samples; (2) clearly diagnosed NEC or sepsis cases with corresponding healthy controls; (3) samples derived from clinically accessible sources (intestinal tissue or blood); (4) datasets with good data quality and standardized preprocessing; and (5) sufficient sample size to allow for differential expression analysis. Following a systematic search of the GEO database, GSE297483, GSE69686, GSE25504 and GSE46619 met all of these inclusion criteria.

For the selected transcriptome data, gene symbols were mapped according to their respective platforms. When multiple probes matched a single gene, the median expression value was used. The expression matrix was normalized using the log2 (X + 1) transformation. After quality control, quantile normalization was performed with the normalizeBetweenArrays function in the limma package to ensure comparable distributions across samples and reduce technical variability.

### 2.3 Pre-selection of diagnostic biomarkers

DEGs analysis was performed in the GSE46619 and GSE25504 datasets using the limma package (Robinson et al., 2009; Ritchie et al., 2015) (for differential expression analysis in RNA sequencing and microarray studies), with a cutoff criterion of P. adj.value <0.05 and |LogFC| > 1. The Benjamini–Hochberg procedure was used to adjust p-values for multiple testing. Overlapping DEGs with the same direction in NS and NEC diseases were identified using a Venn diagram tool. After intersecting the differential genes from both disease datasets, 70 overlapping genes were obtained, of which 69 were upregulated and 1 was downregulated.

### 2.4 GO and KEGG enrichment analysis

GO (Gene Ontology) and KEGG (Kyoto Encyclopedia of Genes and Genomes) enrichment analyses were performed for common driving genes using the clusterProfiler package (Yu et al., 2012) (an R package for comparing biological themes among gene clusters). GO was used to annotate gene biological processes, molecular functions, and cellular components. Gene pathways were annotated through KEGG. Enrichment was considered statistically significant when the P-value was less than 0.05.

### 2.5 Construction of protein-protein interaction networks and screening of hub genes

Overlapping genes were imported into the STRING database (http://string-db.org) (Franceschini et al., 2013) to construct a PPI network with complex interaction relationships (combined score >0.4), which was visualized in Cytoscape (version 3.8.1). Hub genes were identified by scoring differential genes using the degree method and the cytoHubba plugin (Chin et al., 2014). Enrichment analysis and co-expression network analysis of hub genes were performed using the “clusterProfiler” package and GeneMANIA (http://www.genemania.org/) (Warde-Farley et al., 2010), respectively. Molecular Complex Detection (MCODE) plugin in Cytoscape was used to deconstruct functional modules, with selection criteria: degree cutoff of 2, K-core of 2, node score cutoff of 0.2, and maximum depth of 100. Datasets GSE297483 and GSE69686 were used for external validation.

### 2.6 Identification and diagnosis of core genes

Based on the results of external validation, these core genes were successfully identified. To evaluate the diagnostic value of each core gene and multiple genes in NEC and NS, receiver operating characteristic (ROC) curve analyses were performed separately. The area under the ROC curve (AUC) in the GSE297483 and GSE69686 datasets was used to quantify the diagnostic ability of the core genes. The “pROC” R package was used to generate ROC curves (Robin et al., 2011) larger AUC value indicates stronger discriminative ability of the model. In our study, all validated core genes achieved AUC values above 0.7, which suggests at least moderate discriminative ability.

### 2.7 Transcription factor prediction

Transcription regulatory relationships unraveled by sentence-based text mining (TRRUST) were used to obtain candidate transcription factors (TFs) regulating core genes (Han et al., 2017). This database contains rich information about TFs associated with target genes and their regulatory relationships with TFs. We constructed a TF-mRNA regulatory network and visualized it using Cytoscape. For these TFs, we performed internal validation (datasets GSE46619 and GSE25504).

### 2.8 Statistical analysis

Continuous variables were expressed as mean ± standard deviation or median (interquartile range) based on data distribution and compared using Student’s t-test or Mann-Whitney U test. Categorical variables were presented as frequencies (percentages) and analyzed using Chi-square test or Fisher’s exact test. Univariate logistic regression analysis was performed to identify risk factors associated with NEC development, with results presented as odds ratios (OR) with 95% confidence intervals (CI). Variables with *P* < 0.05 in univariate analysis were considered statistically significant. All statistical analyses were conducted using R software (version 4.4.3).

## 3 Results

### 3.1 NS as a risk factor for NEC

This study included 74 infants in the NEC group and 74 infants in the non-NEC group for comparative analysis (Table 1). The two groups showed no significant differences in baseline characteristics or perinatal factors (all *P* > 0.05). In terms of comorbidities, the incidence of feeding intolerance (FI) was higher in the NEC group (82.43% vs. 50.00%, P < 0.001). Laboratory indicators revealed that the platelet count (Plt) in the NEC group was significantly lower than that in the non-NEC group (184.63 vs. 243.00, *P* = 0.002), whereas the white blood cell count (WBC) (13.41 vs. 10.52, *P* = 0.040) and procalcitonin (PCT) levels (0.59 vs. 0.29, *P* < 0.001) were significantly higher (Table 1).

**TABLE 1:** Comparison of baseline characteristics and clinical indicators between NEC and non-NEC groups.

Variable	Variable of factor	NEC_Group (n = 74)	Non_NEC_Group (n = 74)	Statistic	P_Value
Baseline Information					
Gender	girl	32 (43.24%)	34 (45.95%)	0.030	0.869
	boy	42 (56.76%)	40 (54.05%)		
Age at Admission		2.00 (1.00,4.00)	2.00 (2.00,4.00)	−0.702	0.475
BW		1.46 (1.21,1.95)	1.73 (1.35,2.07)	−1.733	0.083
SGA	no	48 (64.86%)	52 (70.27%)	0.280	0.598
	yes	26 (35.14%)	22 (29.73%)		
Perinatal Information					
GA		224.50 (209.00,240.75)	229.50 (214.00,244.50)	−1.212	0.226
Maternal Age		30.00 (27.00,34.00)	30.00 (28.00,33.00)	−0.163	0.872
Multiple Birth	no	54 (72.97%)	55 (74.32%)	<0.001	>0.999
	yes	20 (27.03%)	19 (25.68%)		
History of Abortion	no	51 (68.92%)	48 (64.86%)	0.120	0.727
	yes	23 (31.08%)	26 (35.14%)		
Mode of Delivery	Vaginal Delivery	19 (25.68%)	22 (29.73%)	0.130	0.713
	Cesarean Section	55 (74.32%)	52 (70.27%)		
PROM	no	53 (71.62%)	55 (74.32%)	0.030	0.853
	yes	21 (28.38%)	19 (25.68%)		
Amniotic Fluid Contamination	no	62 (83.78%)	63 (85.14%)	<0.001	>0.999
	yes	12 (16.22%)	11 (14.86%)		
Apgar Score <7 at 1 min	no	53 (71.62%)	63 (85.14%)	3.230	0.072
	yes	21 (28.38%)	11 (14.86%)		
GDM	no	57 (77.03%)	53 (71.62%)	0.320	0.572
	yes	17 (22.97%)	21 (28.38%)		
Preeclampsia	no	54 (72.97%)	57 (77.03%)	0.140	0.704
	yes	20 (27.03%)	17 (22.97%)		
Perinatal Infection	no	66 (89.19%)	65 (87.84%)	<0.001	>0.999
	yes	8 (10.81%)	9 (12.16%)		
Gestational Hypothyroidism	no	69 (93.24%)	68 (91.89%)	<0.001	>0.999
	yes	5 (6.76%)	6 (8.11%)		
Placental Abruption	no	71 (95.95%)	67 (90.54%)	0.970	0.326
	yes	3 (4.05%)	7 (9.46%)		
Comorbidities					
RDS	no	50 (67.57%)	56 (75.68%)	0.830	0.362
	yes	24 (32.43%)	18 (24.32%)		
FI	no	13 (17.57%)	37 (50.00%)	15.980	<0.001
	yes	61 (82.43%)	37 (50.00%)		
Neonatal Anemia	no	7 (9.46%)	21 (28.38%)	7.440	0.006
	yes	67 (90.54%)	53 (71.62%)		
Neonatal Asphyxia	no	53 (71.62%)	60 (81.08%)	1.350	0.246
	yes	21 (28.38%)	14 (18.92%)		
ICH	no	52 (70.27%)	60 (81.08%)	1.800	0.180
	yes	22 (29.73%)	14 (18.92%)		
BPD	no	67 (90.54%)	67 (90.54%)	<0.001	>0.999
	yes	7 (9.46%)	7 (9.46%)		
PDA	no	42 (56.76%)	49 (66.22%)	1.030	0.311
	yes	32 (43.24%)	25 (33.78%)		
Hypoglycemia	no	55 (74.32%)	61 (82.43%)	1.000	0.318
	yes	19 (25.68%)	13 (17.57%)		
Neonatal Pneumonia	no	16 (21.62%)	14 (18.92%)	0.040	0.838
	yes	58 (78.38%)	60 (81.08%)		
Respiratory Failure	no	54 (72.97%)	52 (70.27%)	0.030	0.855
	yes	20 (27.03%)	22 (29.73%)		
Apnea	no	48 (64.86%)	50 (67.57%)	0.030	0.862
	yes	26 (35.14%)	24 (32.43%)		
Neonatal Sepsis	no	29 (39.19%)	49 (66.22%)	9.790	0.002
	yes	45 (60.81%)	25 (33.78%)		
Laboratory Indicators					
ALT		4.50 (3.00,7.15)	4.30 (2.62,6.47)	1.283	0.200
AST		47.70 (32.95,75.38)	49.05 (30.52,69.40)	0.562	0.576
ALP		199.00 (160.25,273.50)	201.50 (147.50,248.25)	0.483	0.630
Plt		184.63 (123.94,258.19)	243.00 (194.25,285.25)	−3.126	0.002
WBC		13.41 (9.95,19.77)	10.52 (8.59,16.38)	2.056	0.040
PCT		0.59 (0.27,5.01)	0.29 (0.18,0.92)	3.632	<0.001
CRP		29.95 (25.50,34.03)	5.63 (3.16,7.09)	10.500	<0.001
Blood Culture	no	53 (71.62%)	73 (98.65%)	19.270	<0.001
	yes	21 (28.38%)	1 (1.35%)		
Treatment Status					
Mechanical Ventilation Use	no	27 (36.49%)	38 (51.35%)	2.740	0.098
	yes	47 (63.51%)	36 (48.65%)		
Dual Antibiotic Use	no	54 (72.97%)	64 (86.49%)	3.390	0.066
	yes	20 (27.03%)	10 (13.51%)		
Blood Product Transfusion	no	10 (13.51%)	24 (32.43%)	6.450	0.011
	yes	64 (86.49%)	50 (67.57%)		
Probiotic Use	no	26 (35.14%)	34 (45.95%)	1.370	0.241
	yes	48 (64.86%)	40 (54.05%)		
Hospital Stay Duration		50.00 (34.00,65.25)	29.50 (17.00,49.00)	4.355	<0.001

Abbreviations: NEC, neonatal necrotizing enterocolitis; BW, birth weight (kg); SGA, small for gestational age; GA, gestational age (days); PROM, premature rupture of membranes; GDM, gestational diabetes mellitus; RDS, respiratory distress syndrome; FI, feeding intolerance; ICH, intracranial hemorrhage; BPD, bronchopulmonary dysplasia; PDA, patent ductus arteriosus; ALT, alanine aminotransferase (U/L); AST, aspartate aminotransferase (U/L); ALP, alkaline phosphatase (U/L); Plt, platelet count (×10^9^/L); WBC, white blood cell count (×10^9^/L); PCT, procalcitonin (ng/mL); CRP, C-reactive protein (mg/L).

Among the 70 infants diagnosed with sepsis, 45 (64.3%) developed NEC while 25 (35.7%) did not. Notably, early-onset sepsis was significantly more prevalent in the NEC group compared to the non-NEC group (57.78% vs. 4.00%, *P* < 0.001), while late-onset sepsis showed the opposite pattern (42.22% vs. 96.00%) (Table 2). Additionally, positive blood cultures were more frequently observed in sepsis patients who developed NEC (46.67% vs. 4.00%, *P* < 0.001).

**TABLE 2:** Comparison of early-onset versus late-onset sepsis in NEC and Non-NEC groups.

Variable	Variable of factor	NEC_Group (n = 45)	Non_NEC_Group (n = 25)	Statistic	P-value
Gender	girl	17 (37.78%)	8 (32.00%)	0.05	0.8235
	boy	28 (62.22%)	17 (68.00%)		
Age at Admission		2.00 (1.00,5.00)	3.00 (2.00,7.00)	−0.8519	0.3878
BW		1.32 (1.15,1.85)	1.41 (1.20,1.87)	−0.1777	0.8637
SGA	no	30 (66.67%)	18 (72.00%)	0.04	0.8478
	yes	15 (33.33%)	7 (28.00%)		
GA		217.00 (207.00,236.00)	221.00 (207.00,231.00)	0.0552	0.9609
ALT		4.40 (2.60,7.00)	4.90 (3.60,6.60)	−0.4474	0.6589
AST		52.00 (31.50,86.30)	52.30 (35.90,68.80)	−0.0429	0.9707
ALP		192.00 (150.00,251.00)	204.00 (171.00,258.00)	−0.8764	0.3841
Plt		169.00 (111.01,239.28)	248.00 (188.00,286.00)	−2.6046	0.0094
WBC		13.41 (9.41,18.83)	12.30 (9.45,20.62)	0.2022	0.8445
PCT		2.78 (0.33,11.38)	0.56 (0.33,2.61)	1.6179	0.1069
CRP		30.98 (25.49,35.04)	5.63 (3.55,6.69)	6.8946	<0.001
Sepsis classification	eos	26 (57.78%)	1 (4.00%)	17.41	<0.001
	los	19 (42.22%)	24 (96.00%)		
Blood Culture	no	24 (53.33%)	24 (96.00%)	11.67	0.0006
	yes	21 (46.67%)	1 (4.00%)		

Abbreviations: NEC, neonatal necrotizing enterocolitis; BW, birth weight (kg); SGA, small for gestational age; GA, gestational age (days); ALT, alanine aminotransferase (U/L); AST, aspartate aminotransferase (U/L); ALP, alkaline phosphatase (U/L); Plt, platelet count (×10^9^/L); WBC, white blood cell count (×10^9^/L); PCT, procalcitonin (ng/mL); CRP, C-reactive protein (mg/L); Eos, Early-onset sepsis; Los, Late-onset sepsis.

**FIGURE 1:**
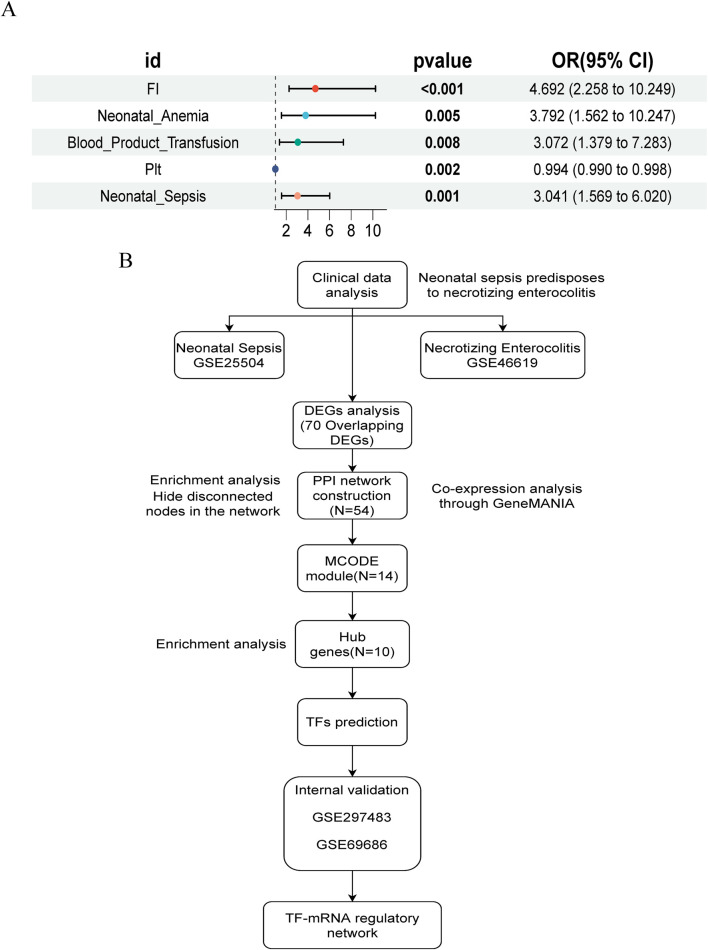
Study design flowchart and NEC risk factor forest map. **(A)** Univariate logistic regression forest plot of NEC risk factors. **(B)** Flowchart of study design and analysis.

In summary, compared to non-NEC infants, those with NEC exhibited more severe systemic inflammatory responses (elevated WBC, PCT, and C-reactive protein (CRP), and decreased Plt), a higher incidence of FI, anemia, and sepsis, increased transfusion requirements, and longer hospital stays. We performed univariate logistic regression analysis for NEC based on statistically significant variables, and the forest plot results indicated that FI, neonatal anemia, blood product use, and NS were risk factors, while Plt levels approached being a protective factor (Figure 1A).

### 3.2 Identification and functional analysis of overlapping DEGs

**FIGURE 2:**
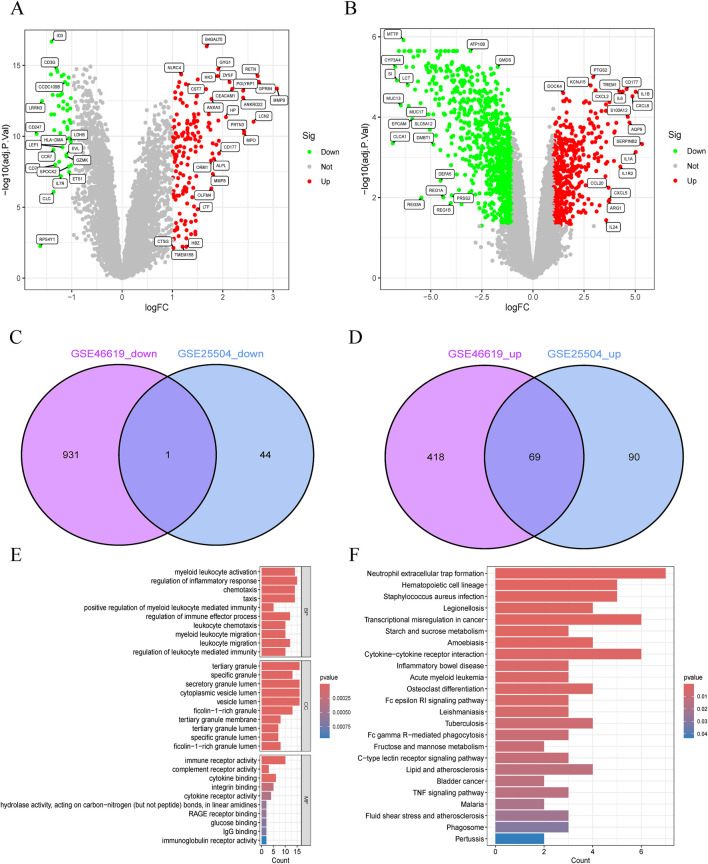
Identification and functional annotation of shared differentially expressed genes (DEGs) in neonatal necrotizing enterocolitis (NEC) and neonatal sepsis. **(A,B)** Volcano plots of DEGs in **(A)** GSE46619 (NEC) and **(B)** GSE25504 (neonatal sepsis) datasets (Red: upregulated; Green: downregulated; Gray: nonsignificant). **(C,D)** Venn diagrams of **(C)** downregulated and **(D)** upregulated DEGs shared between GSE46619 and GSE25504. **(E)** GO enrichment analysis of overlapping DEGs. **(F)** Pathway enrichment analysis of overlapping DEGs.

The study flowchart is illustrated in Figure 1B. A total of 204 and 1420 DEGs were identified from the GSE25504 and GSE46619 datasets, respectively (Figures 2A,B). In the intersection of these datasets, 70 overlapping DEGs with concordant directions of change were identified (69 upregulated and 1 downregulated) (Figures 2C,D). The complete list of DEGs is available as supplementary data, specifically in Supplementary Tables S1-S3. To investigate potential biological functions, GO enrichment analysis and KEGG pathway analysis were conducted on the overlapping DEGs using R software (Figures 2E,F). The results of the GO analysis revealed significant enrichment in the biological process (BP) terms, including cytokine-mediated immune response, leukocyte migration, and positive regulation of immune effector processes. In the cellular component (CC) terms, significant enrichment was observed in extracellular exosomes, specific granule membranes, and granule lumens. Furthermore, in the molecular function (MF) terms, significant enrichment was noted in cytokine receptor binding, immune receptor activity, and integrin binding (Supplementary Tables S4, S5). Additionally, pathways related to pertussis, the tumor necrosis factor (TNF) signaling pathway, cytokine-cytokine receptor interaction, rheumatoid arthritis, acute myeloid leukemia, inflammatory bowel disease, glucose metabolism, and tuberculosis were significantly enriched in the KEGG analysis. These findings suggest that pathways associated with immune response, cytokine signaling, and infection may play crucial roles in the pathogenesis of NS and NEC.

### 3.3 PPI network construction and subnetwork screening

The PPI network contained 43 nodes and 263 interacting pairs (Supplementary Tables S6). An important subnetwork within the hub gene network was screened using the MCODE module in Cytoscape, which included 14 nodes and 80 interacting pairs (Supplementary Tables S7). These 14 nodes were *IL1RN, FPR1, FPR2, HCK, S100A12, CSF3R, IL1B, FCGR1A, FCER1G, CD163, ITGAM, SPI1, MMP9, and C5AR1*, with specific scores detailed in Supplementary Tables S8.

### 3.4 Hub gene selection and enrichment analysis

**FIGURE 3:**
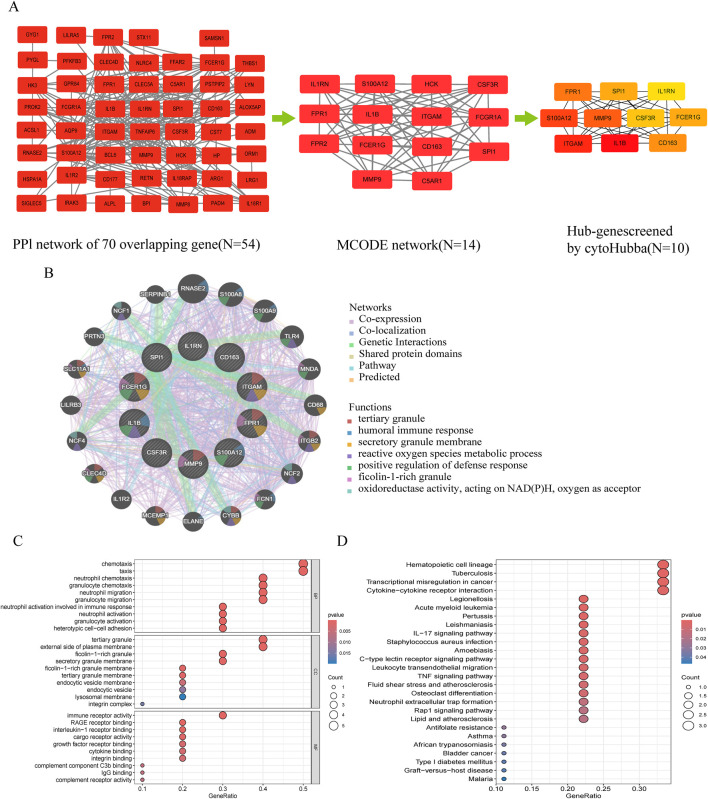
Protein-protein interaction (PPI) network and functional enrichment of shared hub genes in NEC and neonatal sepsis. **(A)** Topological analysis of DEGs: Left: PPI network of shared DEGs (disconnected nodes removed); Middle: Core functional module identified by MCODE algorithm; Right: Hub gene subnetwork. **(B)** Functional features of hub genes in co-expression networks (GeneMANIA analysis, see Supplementary Figure S1 for core functions). **(C)** GO enrichment annotation of hub genes. **(D)** KEGG pathway enrichment of hub genes.

Hub genes were identified using the degree algorithm in the CytoHubba plugin, as detailed in Supplementary Tables S9. Figure 3A presents the decomposition diagram of the PPI network alongside the network constructed from the hub genes. A total of 10 hub genes were identified, including *MMP9, FPR1, FCER1G, CD163, S100A12, ITGAM, SPI1, IL1RN, IL1B, and CSF3R*. The interaction network of these genes was constructed using the GeneMANIA database, revealing significant enrichment in functions related to innate immunity and inflammation, such as tertiary granule formation, humoral immune response, secretory granule membrane, reactive oxygen species metabolic process, positive regulation of defense response, ficolin-1-rich granule, and NAD(P)H oxidoreductase activity (Figure 3B). Subsequent enrichment analysis indicated significant enrichment in immune cell chemotaxis and activation, as well as receptor signaling, particularly in biological processes (e.g., neutrophil/granulocyte chemotaxis, migration, activation, and heterotypic cell adhesion), cellular components (e.g., ficolin-1-rich granules and their membranes, tertiary granules and their membranes, endocytic vesicles and their membranes, lysosomal membranes, integrin complexes), molecular functions (e.g., immune receptor activity, receptor for advanced glycation end products (RAGE) binding, interleukin-1 receptor binding, cytokine binding, integrin binding, complement component C3b binding, IgG binding, complement receptor activity), and KEGG pathways (e.g., cytokine-cytokine receptor interaction, leukocyte transendothelial migration, IL-17 signaling pathway, *Staphylococcus aureus* infection, tuberculosis, pertussis, leishmaniasis, type 1 diabetes) (Figures 3C,D; Supplementary Tables S10, S11).

### 3.5 Diagnostic efficacy of core genes

**FIGURE 4:**
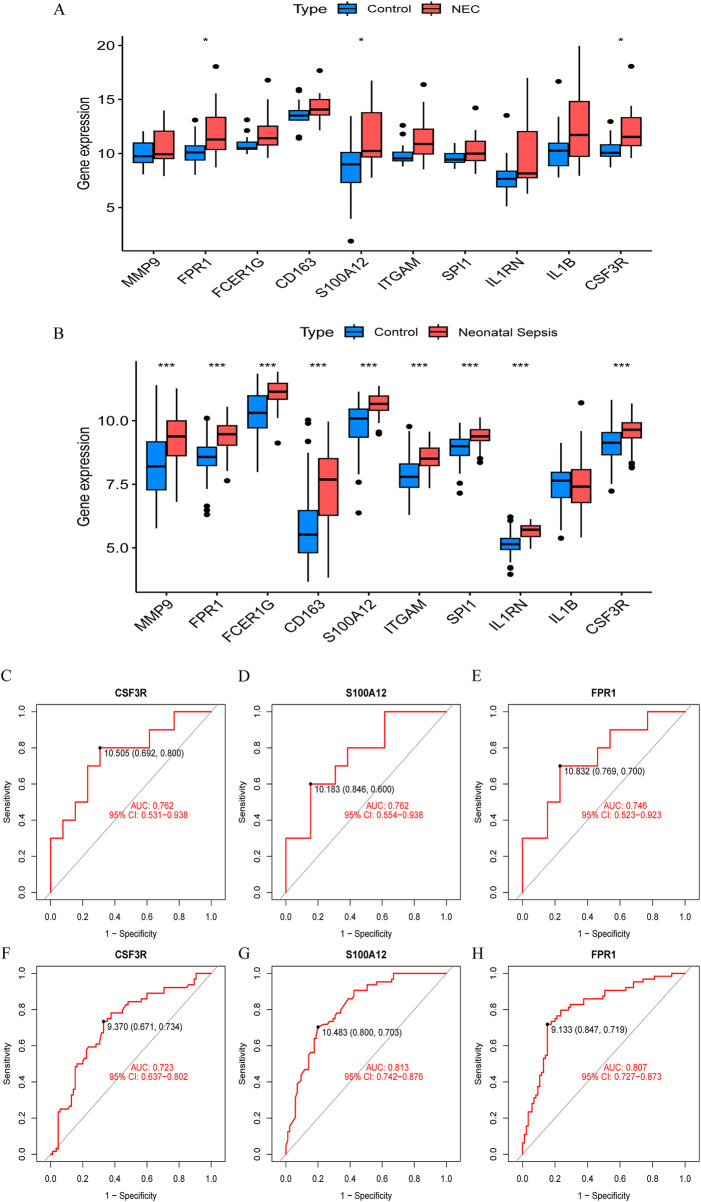
Validation of hub gene expression and diagnostic efficacy in NEC and neonatal sepsis. **(A)** Boxplots of hub gene expression in NEC dataset (GSE297483) vs. healthy controls. **(B)** Boxplots of hub gene expression in neonatal sepsis dataset (GSE69686) vs. healthy controls. **(C–E)** ROC curves for diagnostic biomarkers (CSF3R, FPR1, S100A12) in NEC dataset (GSE297483). **(F–H)** ROC curves for diagnostic biomarkers in neonatal sepsis dataset (GSE69686).

To identify reliable core genes, we conducted external validation on the selected genes. In the NEC dataset (GSE297483), only the expression levels of *FPR1*, *S100A12*, and *CSF3R* were significantly elevated compared to the control group (Figure 4A), while no statistical differences were observed for the other genes. In NS samples, nine genes had significantly higher expression levels than the control group, while *IL1B* showed no significant difference (GSE69686, Figure 4B). Consequently, we identified three core genes: *FPR1*, *S100A12, and CSF3R*. Subsequently, we generated ROC curves using external validation datasets to further assess the diagnostic value of these validated core genes. In the NEC-related validation dataset, the AUC values for *FPR1*, *S100A12*, and *CSF3R* were 0.762, 0.762, and 0.746, respectively (Figures 4C–E). Furthermore, in the NS-related validation dataset, the AUC values for all validated core genes exceeded 0.7, measuring 0.723, 0.813, and 0.807, respectively (Figures 4G–I).

### 3.6 Integrated TF-mRNA network

**FIGURE 5:**
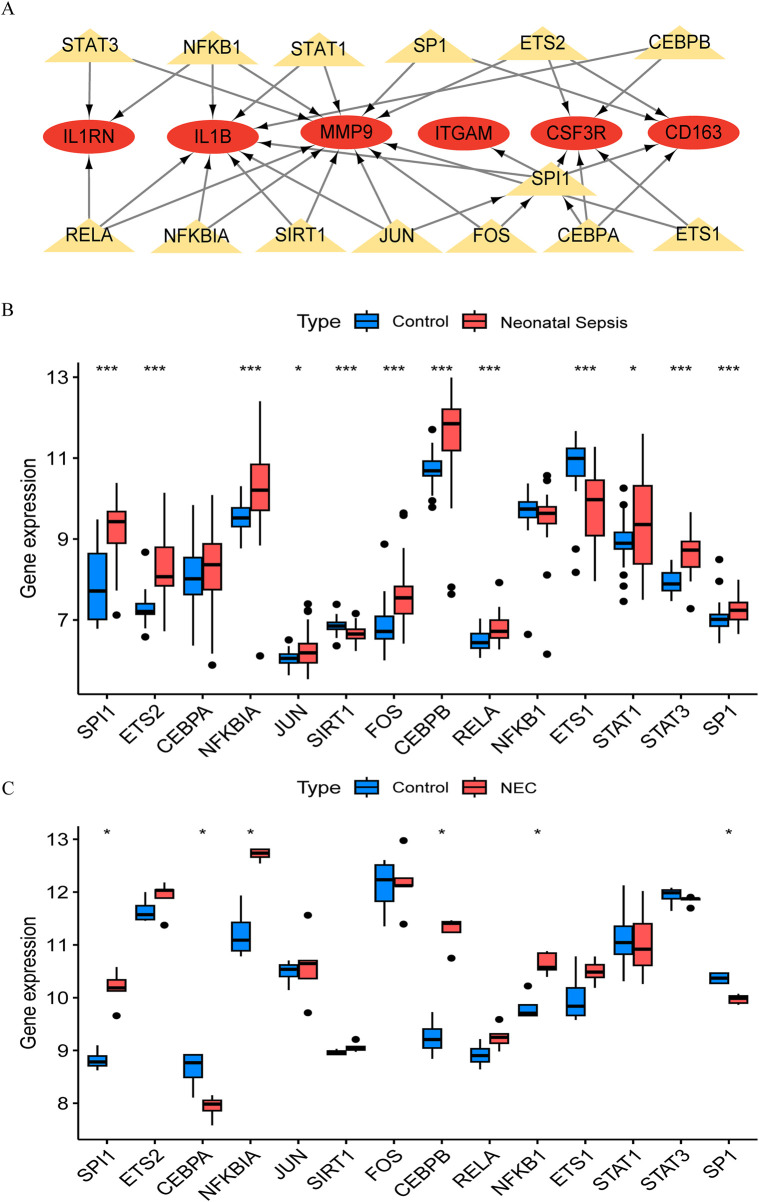
Transcriptional regulatory network and validation in NEC and neonatal sepsis. **(A)** Hub gene-transcription factor (TF) interaction network (Red ovals: DEGs; Yellow triangles: TFs). **(B)** Expression of core TFs in NEC validation cohort (GSE46619) vs. healthy controls. **(C)** Expression of TFs in neonatal sepsis validation cohort (GSE25504) vs. healthy controls.

The TRRUST database provides insights into the regulatory relationships between transcription factors and their target genes, elucidating their interactions. In this study, we utilized transcription factor binding site information from TRRUST to identify key transcription factors and target genes associated with NEC and NS. We identified a total of 35 associations involving 14 transcription factors (*SPI1, ETS2, CEBPA, NFKBIA, JUN, SIRT1, FOS, CEBPB, RELA, NFKB1, ETS1, STAT1, STAT3, and SP1*) and 6 core genes (*IL1B, ITGAM, CSF3R, CD163, MMP9,* and *IL1RN*), with specific regulatory relationships detailed in Supplementary Tables S12. Based on these findings, we constructed a transcription factor-messenger RNA regulatory network using Cytoscape software (Figure 5A) and validated it against the NEC and NS datasets. By analyzing gene expression data from the GSE46619 (NEC) and GSE25504 (NS) datasets, we confirmed the expression changes of these transcription factors in both conditions. Validation results indicated that several transcription factors exhibited significant expression changes in NEC and NS, further supporting their putative regulatory roles in these diseases (Figures 5B,C). We distinguished mRNA and TFs through different shapes and colors, effectively illustrating this regulatory network and suggesting that these TFs may play crucial roles in the pathological processes of NEC and NS.

## 4 Discussion

This study explored the potential relationship between neonatal sepsis and NEC development through integrated clinical and bioinformatics approaches. Our clinical analysis suggests that sepsis may serve as a risk factor for NEC, with affected infants demonstrating heightened inflammatory responses and increased healthcare complexity. The bioinformatics investigation identified shared molecular signatures between these conditions, particularly involving genes related to neutrophil function and inflammatory processes (*FPR1, S100A12, CSF3R*). These findings suggest that common inflammatory pathways may underlie the clinical association between sepsis and NEC, providing insights into potential mechanisms linking these conditions in neonatal populations.

Our clinical data suggest that sepsis may serve as a risk factor for NEC, consistent with previous observations. Early-onset sepsis was more prevalent in NEC patients, suggesting that early inflammatory insults may predispose to intestinal complications, consistent with known effects of systemic inflammation on gut barrier function. The pathophysiological connection may involve several mechanisms. Neonatal gut microbiota dysbiosis can compromise mucosal barrier function, facilitating pathogen colonization and subsequent systemic inflammation (Garvey, 2024; Kamble et al., 2024). Sepsis-induced inflammatory responses, particularly through TLR4 pathway activation, may exacerbate intestinal immune imbalance and epithelial damage (Li et al., 2024). Premature infants appear particularly vulnerable due to insufficient expression of immune regulatory factors such as TOLLIP and SIGIRR (Gomart et al., 2021). The clinical presentation in our NEC cohort reflected these inflammatory processes, with elevated inflammatory markers and thrombocytopenia correlating with disease severity. Additional factors such as neonatal anemia and transfusion requirements may further contribute to intestinal injury through tissue hypoxia and transfusion-associated inflammatory responses (Dang et al., 2024). These overlapping systemic features between NEC and NS provided the rationale for our bioinformatics investigation of shared molecular mechanisms.

The identification of 70 overlapping DEGs with conserved expression patterns between NS and NEC datasets suggests potential shared pathological mechanisms. The enrichment of these genes in immune-related pathways, particularly cytokine signaling and leukocyte activation, may reflect the characteristic immune dysregulation observed in both conditions. This pattern appears consistent with known features of neonatal immune immaturity (Sanidad and Zeng, 2020), including reduced monocyte sensitivity to TLR ligands and impaired dendritic cell IL-12p70 secretion (Wang et al., 2023). The mechanistic implications of these findings deserve consideration. Cytokine-cytokine receptor interactions, particularly involving TNF-α and IL-17 pathways, may contribute to NF-κB activation and subsequent intestinal barrier dysfunction through downregulation of tight junction proteins (Nie et al., 2024). The involvement of inflammatory amplification mechanisms, including sympathetic nerve-mediated immune cell infiltration, could potentially explain the progression to intestinal necrosis characteristic of NEC (Tanaka et al., 2023). These observations suggest that select overlapping DEGs, warrant investigation as potential biomarkers for both conditions.

The PPI network analysis suggests that neutrophil-related genes (*FPR1, S100A12, CSF3R, ITGAM*) and inflammatory mediators (*IL1B, MMP9, IL1RN*) may function coordinately in sepsis-complicated NEC. These genes appear to participate in processes involving damage sensing, chemotactic recruitment, and cellular activation. During the inflammatory amplification phase, *IL-1*β may induce pro-inflammatory factor expression through NF-κB pathway activation, with insufficient IL1RN expression potentially exacerbating this response (Kaminsky et al., 2021). MMP9 could disrupt tight junction proteins and upregulate IL-8 signaling, forming a positive feedback loop that compromises barrier function (Mariaule et al., 2021). Clinical observations supporting this model include the positive correlation between neutrophil dysfunction and NEC surgical risk (Sokou et al., 2025), and the association between reactive oxygen species (ROS) levels and epithelial apoptosis rates in NEC tissues (Mariaule et al., 2021). This integrated model suggests that bacterial infection may trigger *FPR1/CSF3R* signaling, leading to neutrophil infiltration and subsequent *IL-1*β*/MMP9* release, ultimately resulting in intestinal barrier disruption and bacterial translocation (Wenceslau et al., 2019; Al-Sadi et al., 2021). While this framework provides mechanistic insights into sepsis-NEC progression, experimental validation remains necessary to confirm these proposed relationships.

The consistent upregulation of *FPR1*, *S100A12*, and *CSF3R* in both NEC and NS validation cohorts highlights their potential as clinically relevant biomarkers. Among these, *S100A12* is particularly promising. Studies have demonstrated that serum *S100A12* is a highly sensitive and specific biomarker for neonatal sepsis, outperforming conventional markers like CRP in early detection (Tosson et al., 2019). Furthermore, fecal *S100A12* levels are significantly elevated 4–10 days before the clinical onset of severe NEC, offering a critical window for early risk assessment and intervention (Desorcy-Scherer et al., 2021). *S100A12* may contribute to inflammation through TLR4/NF-κB signaling, potentially associated with intestinal mucosal changes (Cao et al., 2024).

As a primary sensor for bacterial and mitochondrial N-formyl peptides, *FPR1* orchestrates neutrophil chemotaxis and activation, a process central to the pathogenesis of both infection-driven sepsis and injury-associated NEC (Kwon et al., 2024). While essential for pathogen clearance, this *FPR1*-driven neutrophil response can also inflict severe collateral tissue damage, making it a double-edged sword in the fragile neonatal environment (Tarassishin et al., 2025). Therefore, *FPR1* not only holds potential as an early biomarker for inflammatory crises but also represents a promising therapeutic target for modulating the destructive hyper-inflammation characteristic of these devastating neonatal diseases.

*CSF3R* regulates neutrophil proliferation, differentiation, and survival through granulocyte colony-stimulating factor (G-CSF) signaling (Liu et al., 2023). During neonatal infections, increased *CSF3R* expression is associated with enhanced innate immune responses. However, dysregulated *CSF3R* signaling may lead to the release of immature neutrophils, which could reduce microbial clearance efficiency and contribute to tissue damage. These findings suggest that *CSF3R* might function both as an indicator of inflammatory intensity and a candidate target for therapies aimed at improving neutrophil function.

Our transcription factor analysis identified *SPI1, NFKB1*, and *JUN* as potential regulatory factors in the NS-NEC pathway, though the mechanistic relationships require careful interpretation. *SPI1* may influence *CSF3R* expression, potentially affecting neutrophil function through *JAK/STAT* signaling (Zhang et al., 2024). The *NFKB1-IL1B* regulatory relationship suggests possible involvement in inflammatory amplification, as *NF-κB* signaling has been implicated in cytokine production and intestinal barrier dysfunction (Pozzi et al., 2023). *JUN* appears to regulate *MMP9* expression, which could contribute to tissue remodeling processes, though the specific role in neonatal intestinal pathology remains to be fully characterized (Zhu et al., 2025). While these transcription factors may work collectively to influence immune cell activation and tissue responses, the extent of their contribution to sepsis-complicated NEC pathogenesis requires further investigation. The complex interplay between these regulatory networks suggests that therapeutic targeting of individual pathways may have limited efficacy, highlighting the need for more comprehensive approaches to understanding and treating these conditions.

## 5 Limitations

This study has certain limitations that need to be addressed in future research. First, as a retrospective study, although the sample size is large and externally validated, potential biases may still exist, requiring further validation through prospective studies. Second, the identified core genes have not been functionally validated in humans. Although their functions have been predicted through bioinformatics, their specific roles in NEC and NS still require *in vitro* cell experiments and *in vivo* animal model validation. Finally, the study mainly focused on gene expression and bioinformatics analysis. The specific molecular mechanisms by which core genes regulate immune responses still need to be elucidated through detailed molecular biology experiments.

## 6 Conclusion

This study suggests that neonatal sepsis may serve as a risk factor for NEC development through shared inflammatory pathways. Our integrated analysis identified overlapping gene expression patterns between NS and NEC, with *FPR1*, *S100A12*, and *CSF3R* showing potential diagnostic utility across both conditions. These genes appear to participate in inflammatory processes involving immune-cell recruitment and activation, possibly mediated by transcriptional networks including *SPI1*, *NFKB1*, and *JUN*. While these computational findings provide insights into potential common mechanisms between NS and NEC, prospective clinical validation and functional experiments are necessary to confirm these relationships and assess their therapeutic implications.

## Data Availability

The datasets presented in this study can be found in online repositories. The names of the repository/repositories and accession number(s) can be found below: https://www.ncbi.nlm.nih.gov/, GSE25504 https://www.ncbi.nlm.nih.gov/, GSE46619 https://www.ncbi.nlm.nih.gov/, GSE297483 https://www.ncbi.nlm.nih.gov/, GSE69686.

## Glossary

NEC: Necrotizing Enterocolitis
NS: Neonatal Sepsis
EOS: Early-onset sepsis
LOS: Late-onset sepsis
DEGs: Differentially Expressed Genes
PPI: Protein-Protein Interaction
GO: Gene Ontology
KEGG: Kyoto Encyclopedia of Genes and Genomes
TF: Transcription Factor
ROC: Receiver Operating Characteristic
AUC: Area Under the Curve
CRP: C-reactive protein
PCT: Procalcitonin
WBC: White Blood Cell Count
Plt: Platelet Count
SGA: Small for Gestational Age
RDS: Respiratory Distress Syndrome
PDA: Patent Ductus Arteriosus
FI: Feeding Intolerance
ICH: Intracranial Hemorrhage
BPD: Bronchopulmonary Dysplasia
ALT: Alanine Aminotransferase
AST: Aspartate Aminotransferase
ALP: Alkaline Phosphatase
BP: Biological Process
CC: Cellular Component
MF: Molecular Function
TNF: Tumor necrosis factor
TLR4: Toll-like receptor 4
ROS: Reactive Oxygen Species
RAGE: Receptor for advanced glycation end products
G- CSF: Granulocyte Colony-stimulating Factor
MCODE: Molecular Complex Detection
